# Neuroendoscopic aspiration and lavage of intraventricular empyema following shunt infection in infants

**DOI:** 10.11604/pamj.2018.31.15.16631

**Published:** 2018-09-05

**Authors:** Steven Tandean, Lutfi Hendriansyah, Stanislaus Djokomuljanto, Nico Adi Saputra, Andry Juliansen, Silvia Valentina, Monica Ann Mulry, Julius July

**Affiliations:** 1 Department of Neurosurgery, Faculty of Medicine, Pelita Harapan University, Neuroscience Centre Siloam Hospital Lippo Village, Tanggerang, Indonesia; 2 Department of Neurosurgery, Faculty of Medicine, Universitas Sumatera Utara, Medan, Indonesia; 3 Department of Pediatric, Siloam Lippo Karawaci Hospital, Tanggerang, Indonesia; 4 Faculty of Medicine, Pelita Harapan University, Tanggerang, Indonesia; 5 Undergraduate School of Medicine National University of Ireland, Galway, Ireland

**Keywords:** Neuroendoscopic, lavage, intraventricular empyema, shunt infection, infant

## Abstract

Bacterial ventriculitis is one of the most common and serious complications of shunt placement. Shunt infection has varied management and is difficult to treat neurosurgically. We report a case of intraventricular empyema due to shunt infection. Standard management was failed for this case and reaccumulation of pus in the both ventricles. Neuroendoscopic surgery with intraventricular lavage and aspiration using cannula nasogastric tube (NGT) through a single burr hole, has successfully decreased the accumulation of intraventricular empyema. After lavage and aspiration, antibiotic can be distributed effectively to the affected area. Follow up imaging and cerebrospinal fluid (CSF) culture shown a good result and shorter length of stay in the hospital. Neuroendoscopy appears effective and safe for the management of bacterial ventriculitis due to shunt infection in infant. The strategy described in this report might be useful to treat intraventricular empyema.

## Introduction

One of the most common and serious complications of ventriculoperitoneal (VP) shunt is infection. Although the shunt infection is high, management of shunt infection is still not optimal with shunt infection management protocols varying significantly between centers. Multiple review articles suggest ideal management which consists of antibiotics, complete shunt removal with external ventricular drain (EVD), and reimplantation of VP shunt after cerebrospinal fluid (CSF) sterilization. Several reports also suggested intrathecal antibiotic administration or intraventricular irrigation, but its practical applications are controversial [1]. We report a case of the effectiveness of endoscopic aspiration and lavage for intraventricular empyema after ideal management failed.

## Patient and observation

**Figure 1:**
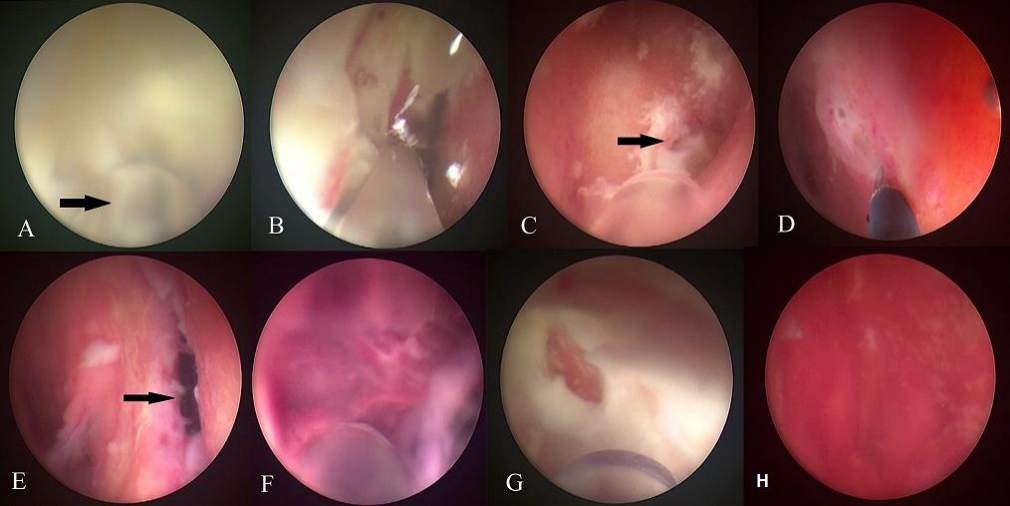
(A,B) intraoperative findings and surgical procedures. Thick pus in the ipsilateral ventricle, NGT 8 Fr was used as a cannula suction (arrow); (C) foramen monro filled by pus; (D,E) septostomy was performed using monopolar; (F) blood clots and pus in the contralateral ventricle; (H) after pus and blood clot are removed, the wall of the ventricle shows an inflammation

A two months old boy was referred from other hospital with fever and meningeal signs due to ventriculitis. From history taking revealed he had cele resection due to ruptured and VP shunt. The shunt system was infected and removed immediately then replaced with EVD. Culture of CSF and catheter showed enterobacter cloacae. Patient was hospitalized for 14 days with twice EVD procedure and administration of intrathecal antibiotic before referred to our hospital. The MRI brain performed showed persistent ventriculitis including intraventricular empyema of both lateral ventricles with evidence restriction on diffusion-weighted imaging (DWI). The aforementioned treatment was not effective in treating the accumulation of empyema in the both ventricles. Severe pus and debris caused uneven drainage and intrathecal antibiotics, so empyema aspiration and irrigation using neuroendoscopy was considered. This method used LOTTA^®^ ventriculoscope (Karl Storz-Endoskope, Germany) to active aspirate and extensive ventricular irrigation using warm normal saline. This ventriculoscope has two separate channels to be used simultaneously, one channel used for normal saline irrigation and other channel for suction using nasogastric tube 8 Fr. A right frontal burr hole from the previous surgery was used for the neuroendoscopic approach. Multiple deposits of thick yellow colored pus, blood clots and inflammatory debris of ventricle walls were observed in the lateral ventricle. Continuous irrigation with warm normal saline and active aspiration of the pus using nasograstric tube (NGT) was carried out. After ipsilateral ventricle was cleared of infective material, septostomy was performed. The ventriculoscope was inserted to the contralateral ventricle through the hole from septostomy and then same procedure was performed. The pus was removed using suction and floating materials were washed out by continuous irrigation. The procedure was done with placement of EVD for evaluation and administration of intrathecal antibiotic (Figure 1). After the procedure, patient was administrated Amikacin IV 100mg daily and Amikacin intrathecal 10 mg daily based on culture sensitivity for Enterobacter cloacae. CSF analyze during follow up showed normal characteristics. Brain MRI with DWI sequence on the day 5 after surgery showed significant improvement without restriction. The patient's condition improved clinically, and CSF culture was negative. After 3 day with dependent test, DWI imaging was taken. The imaging showed no restriction and the ventricle size has no significant difference. Patient was discharged within 10 days hospitalization with good condition and follow up fontanelle and head circumference regularly. VP shunt was performed 2 months after discharged to reduce ventricle size. Patient was discharged 5 days after VP shunt with good outcome.

## Discussion

Purulent brain abscess cause by bacterial ventriculitis still a main cause of morbidity and mortality in patients with an infected shunt. MRI has an important role as a standard diagnostic imaging, especially DWI sequence. DWI has a high sensitivity for detecting intraventricular debris and pus, which are the most frequent sign of ventriculitis. DWI sequence can also be used to evaluate effectiveness of any treatment or procedure [2]. Many articles on the topic of shunt infection suggest ideal management consist of antibiotics, complete shunt removal with placement of EVD, and reimplantation with VP shunt after evidence of CSF sterilization. A survey also showed that most board-certified members of the American Society of Pediatric Neurosurgeons treat ventricular shunt with this method. Intrathecal antibiotic administration is still a controversial, although theoretically make sense because of enhanced antibiotic concentration in CSF. The most potential adverse effect of intrathecal antibiotics is neurotoxicity. Indication for intrathecal antibiotic administration is still not established. Administration of intrathecal antibiotic is usually indicated for persistent positive cultures after systemic antibiotics or specific bacteria, like gram-negative infections are identified as the cause of infection. However, ventriculitis can still persist despite VP shunt removal and commencement of intravenous and/or intrathecal antibiotics. For cases like this, further treatment options should be considered [1, 3]. Intraventricular drainage in our case failed. This might be due to the thickness of the pus and debris that almost completely filled both lateral ventricles. Severe pus and debris caused uneven irrigation and intrathecal antibiotics couldn't effectively reach the affected areas. Nevertheless, this condition may cause catheter related infection, which would aggravate the infection in the ventricles. Risk of aggravating bacterial ventriculitis by retrograde infection because of excessive and long-term external drainage of CSF should be avoided. James and Bradley found that CSF shunt infection treated with intravenous antibiotics and intrathecal antibiotic via EVD, has a total hospital stay of 15.2 ± 2.3 days. In our case, management of intraventricular empyema using endoscopic aspiration and lavage had a shorter length of stay, just 10 days [4].

There have been previous reports of neuroendoscopic surgery utilization to treat bacterial ventriculitis. Tabuchi and Kadowaki [5] reported two cases with ventriculitis and progressive hydrocephalus after shunt infection can be successfully treated by neuroendoscopic septostomy with intraventricular irrigation using single burr hole. Wang et *al*. [6] reported 41 cases of pyogenic cerebral ventriculitis treated with neuroendoscopic surgery combined with modified EVD when debris and bacterial plaques could not be removed completely, and it showed a relatively favorable outcome. Kumar et *al*. [7] also reported the role of endoscopic lavage in improving the outcome of 8 patients with multidrug resistant gram-negative ventriculitis. Terada et *al*. [8] compared treatment of ventriculitis with neuroendoscopic ventricular irrigation (6 cases) and conventional treatment (8 cases). Result of the report showed shorter duration of EVD placement and better clinical outcome (mRS≤3). Preterm and term newborn infants are fragile, but neuroendoscopic surgery can be performed safely and is a viable treatment option in ventriculitis and intraventricular hemorrhage. Schulz et *al*. demonstrated the possibility and safety of neuroendoscopic procedure in neonates with 20 endoscopic procedures were performed in 14 infants, of which 10 procedures were endoscopic lavage for ventriculitis and intraventricular hemorrhage. Reports concluded with positive outcomes may be achieved for ventriculitis and intraventricular hemorrhage cases in neonates. Neuroendoscopic surgery can be considered as an effective for the treatment of ventriculitis caused by shunt infection [9]. The procedure in this study was using ventriculoscope with two separate channels used simultaneously. One channel was used for continuous warmed normal saline irrigation (around 37°C) and other channel for suction using nasogastric tube 8 Fr. Suction was placed in off position and only switched on during irrigation or for suction of the pus or blood clot. The procedure was ceased when the CSF has a clear appearance without any active bleeding. Report by Brusius and Cavalheiro showed usage of warmed normal saline was safe for irrigation in neonates and also has haemostatic effect if hemorrhage were to occur during the procedure [10]. There are two suggestions for the optimal timing for neuroendoscopic procedure. Two reports suggest procedure should be performed around 2 weeks after aggressive intravenous antibiotic treatment with unsatisfactory effect [5, 9]. Another report suggests performing the procedure immediately after ventriculitis diagnosis for an improved prognosis [9]. We propose neuroendoscopic procedure should be performed immediately after diagnosis of ventriculitis since pus and inflammatory debris can disrupt distribution of antibiotic administration given by intravenously and/or intrathecally.

## Conclusion

Endoscopic aspiration and lavage is effective for the treatment of intraventricular empyema after ideal management failed.

## Competing interests

The authors declare no competing interest.
